# Nucleoproteins of Negative Strand RNA Viruses; RNA Binding, Oligomerisation and Binding to Polymerase Co-Factor

**DOI:** 10.3390/v2010027

**Published:** 2010-01-07

**Authors:** Rob W.H. Ruigrok, Thibaut Crépin

**Affiliations:** Unit for Virus Host Cell Interactions, UMI 3265 UJF-EMBL-CNRS, BP 181, Grenoble cedex 9, France

## Abstract

Commentary on Tawar, R.G.; Duquerroy, S.; Vonrhein, C.; Varela, P.F.; Damier-Piolle, L.; Castagné, N.; MacLellan, K.; Bedouelle, H.; Bricogne, G.; Bhella, D.; Eléouët, J.-F.; Rey, F.A. Crystal structure of a nucleocapsid-like nucleoprotein-RNA complex of respiratory syncytial virus. *Science* **2009**, *326*, 1279–1283.

RSV is a non-segmented, negative strand RNA virus (family: *Paramyxoviridae*; order: *Mononegavirales*). The viral RNA of this group of viruses is always bound to the viral nucleoprotein (N) and this N-RNA structure is the template for viral transcription and replication by the viral RNA-dependent RNA polymerase complex consisting of L (Large protein; actual polymerase) and P (Phosphoprotein; polymerase co-factor). In a recent article in *Science*, Tawar and coworkers published the crystal structure of the RSV nucleoprotein in complex with cellular RNA [1]. All the N-RNAs of the viruses in the *Mononegavirales* order form helical structures and, because these are very flexible, they are not simple objects for structural biology studies. Fortunately, recombinant nature has helped a bit; when the nucleoproteins of vesicular stomatitis virus (VSV), rabies virus and RSV were expressed in bacteria or insect cells, they bind to short cellular RNAs (possibly tRNA molecules) and form short N-RNA complexes that close up into rings through protein-protein contacts [1–3]. When such circular recombinant complexes are separated into size classes that contain only one type of ring, with for instance only 10 or 11 nucleoprotein protomers per ring, they can be crystallised and their atomic structure solved. It is expected that the structure of the nucleoprotein in such N-RNA rings is the same as in the helical nucleocapsid present in the virion. However, due to the constraints of the rings, they do not have the flexibility necessary for biological activity.

Figure 1A shows the structure of a single protomer of rabies virus nucleoprotein in contact with nine nucleotides of cellular RNA. The rabies virus N-RNA rings that were crystallised contained 11 protomers of N plus 11 times nine nucleotides [2]. The VSV N-RNA rings contained 10 protomers plus 10 times nine nucleotides but the structure of the protein and the mode of RNA binding are identical to those of rabies virus N-RNA [3]. Figure 1B shows the structure of RSV N in contact with seven nucleotides. The RSV N-RNA rings contained 10 N protomers plus 10 times seven nucleotides [1]. Figure 1C and D show the structures of the Borna disease virus (BDV) nucleoprotein that was crystallised without RNA [4] and that of the influenza virus nucleoprotein (NP) also without RNA [5]. BDV is also part of the *Mononegavirales* order but influenza virus is a segmented negative strand RNA virus. The nucleoproteins of rabies virus, RSV and BDV have a conserved helical C-terminal domain (top domain in the structures in Figure 1A–C) and a more variable N-terminal domain. For the rabies virus, VSV, and RSV structures, the RNA is bound in a basic groove between the C- and N-terminal domains (bottom row in Figure 1). For BDV it was recently shown that mutation of basic residues lining this groove abolishes RNA binding [6], suggesting a conserved mode of RNA binding. The structure of the influenza virus nucleoprotein differs substantially from that of the other three; there are no C- or N-terminal domains as the chain crosses several times from top to bottom and back. There is no narrow RNA binding groove on influenza virus NP but a large positively charged surface that has been shown by mutational analysis to be involved in RNA binding [7]. This surface is much larger than the grooves of the other proteins because influenza virus NP can fix 24 nucleotides [8], many more than the other proteins (nine for rabies virus and VSV, seven for RSV; the number is not known for BDV). The mode of RNA binding by rabies virus N (identical also for VSV) is remarkably similar to that of RSV N (Figure 2): In the middle of the protein three stacked nucleotide bases bind in a cavity formed on the protein surface (green bases in Figure 2). For RSV, these three nucleotides are consecutive (nucleotides 4–6) whereas for rabies virus a single nucleotide base (5, red) is looped out, leaving the remaining three stacked (4, 6 and 7). The rest of the nucleotides (yellow) bind with their sugar phosphate side to the protein and the bases pointing away. For rabies virus, nucleotide base one stacks onto base nine of the neighbouring N protomer making a stack of five bases (yellow and grey) crossing the N-N interface. For RSV, nucleotide base one stacks onto base seven of the next protomer, forming a stack of four bases (yellow and grey) crossing the N-N interface. The major difference between the rabies virus N-RNA rings and those of RSV is that the RNA is inside the rabies virus rings but outside the RSV rings. It is not known whether this is a significant biological or structural difference or whether this may be due to steric constraints of the rings.

The rabies virus and RSV nucleoproteins have extended N-terminal strands (in blue in Figures 1A and B) that bind onto or insert into the neighbouring N protomer forming an important protein-protein contact in the N-RNA structure. Because the BDV N-RNA structure is not known, we do not know if the “blue” strand of N performs the same function in the N-RNA complex. For influenza virus NP, it is not an N-terminal strand but a β-hairpin towards the C-terminal end of the molecule that inserts into the neighbouring protomer (in green in Figure 1D). Rabies virus, RSV and BDV nucleoproteins also have C-terminal extensions (in red in Figures 1A–C). For RSV, this extension consists of a part of random coil, a poorly organised helix whereas the last 12 to 20 amino acids are disordered (Figure 1B). For rabies, it consists of a part of random coil, a helix, a disordered part that is invisible in the structure (dotted in Figure 1A) and it ends with a helical part that comes back to and binds onto the main body of the C-terminal domain. For measles and Sendai virus, two paramyxoviruses that belong to another genus of the *Paramyxoviridae*, the nucleoprotein has an even longer and natively disordered C-terminal tail, called N_TAIL_ [9]. A sequence in the middle of N_TAIL_ forms an α-helical molecular recognition element that consists of a dynamic and interchanging population of overlapping helices [10]. The viral RNA-dependent RNA polymerase complex binds to the N-RNA with the help of P. For measles and Sendai virus, the C-terminal end of P forms a very small and dynamic three-helical bundle that stabilises when it binds to the helical recognition element in N_TAIL_ [10–12]. Therefore, it is possible that the poorly formed helix in the C-terminal domain of RSV N constitutes the binding site of RSV P. The C-terminal domain of rabies virus (and VSV) P that binds to N is much larger and more structured than those of measles and Sendai virus P [13,14]. The rabies virus P domain binds to the top of the C-terminal domain of N. The “red” loops of the same N protomer plus that of the neighbouring N fold onto and enclose the C-terminal domain of P on two sides [15,16]. Therefore, despite the fact that the structures of the C-terminal domains of rabies virus and VSV P look very different from those of measles and Sendai virus, and despite the fact that the C-terminal domains of N they bind to are not conserved, the binding mode of P to an unfolded domain of N, which then folds upon binding, is very similar. Influenza virus does not have a phosphoprotein polymerase co-factor and does not have a domain equivalent to the “red” domains in the rabies virus and RSV structures.

Apart from opening up the possibility of developing new drugs against RSV, the structure of the nucleocapsid-like RSV complex also clearly shows what is conserved in the nucleoproteins of the negative strand RNA viruses and what is not. Because the conservation of structure and RNA binding is now so clearly shown, it becomes even more obvious how different the influenza virus NP is. Finally, all those who have been working on the structure and function of Sendai and measles virus transcription and replication know that the nucleoproteins of these viruses bind six nucleotides [17–19] and that some biological activities depend on the exact position in which a specific nucleotide is bound to the nucleoprotein [20]. Although a “rule of six” has never been shown for the RSV nucleocapsid, most of us expected that the RSV protein would also bind six nucleotides. So now we know that it does not, showing that one should not assume things but measure them.

## Figures and Tables

**Figure 1.**
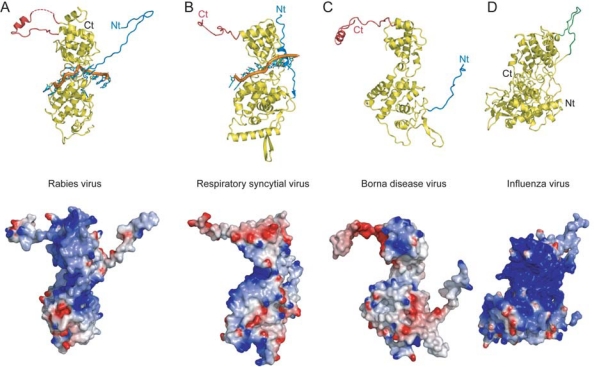
Four structures of negative strand RNA virus nucleoproteins. Ribbon diagrams (Top) and electrostatic surfaces (bottom) of the structures of: **A**: the rabies virus nucleoprotein with bound RNA (PDB code 2GTT). For clarity 11 ribonucleotides are shown, although each protomer only binds nine nucleotides. The ribbon diagram shows a C-terminal top domain and an N-terminal bottom domain. The N-terminal exchange domain is shown in blue and the C-terminal domain involved in binding to P is shown in red. For the electrostatic surface potential shown in the bottom row, the RNA was removed. The potential of this and the other structures goes from −5kT/e (red) to +5kT/e (blue). **B**: the RSV nucleoprotein with bound RNA (PDB code 2wj8). For clarity, nine nucleotides are shown although each protomer only binds seven nucleotides. The orientation and colour coding is the same as for A. For the electrostatic surface potential the RNA was removed as in A. **C**: the BDV nucleoprotein that was crystallised without RNA (PDB code 1N93). Same orientation and colour coding as for A and B although the functions of the N- and C-terminal domains have not yet been established in the context of the N-RNA complex. **D**: the influenza virus nucleoprotein (PDB code 2QO6). This nucleoprotein does not have C- or N-terminal domains. The β-hairpin involved in domain exchange is indicated in green.

**Figure 2.**
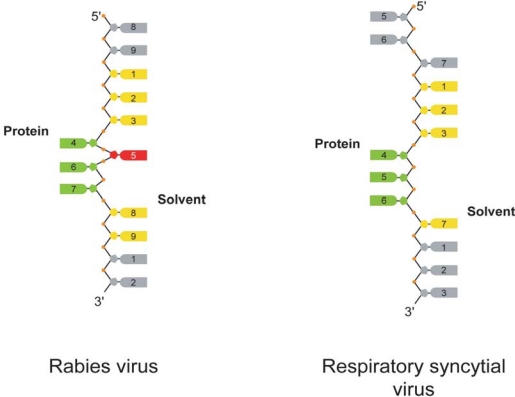
Binding of RNA to the rabies virus and RSV nucleoproteins. The green nucleotide bases bind in a pocket on the protein surface whereas the yellow bases point away from the protein. The red base of the RNA bound to the rabies virus N also points away from the protein so that the three remaining green bases can stack. The grey nucleotides are on the neighbouring N protomers. For comparison, the nucleotides are numbered from 5′ to 3′ whereas Tawar *et al*. had numbered these from 3′ to 5′ [1].
